# Puerarin alleviates sleep disorders in aged mice related to repairing intestinal mucosal barrier

**DOI:** 10.1007/s13659-023-00390-3

**Published:** 2023-09-12

**Authors:** Qing Tao, Jinhua Zhang, Qiao liang, Shiyu Song, Shuxia Wang, Xiaoming Yao, Qian Gao, Lei Wang

**Affiliations:** 1 Center for Translational Medicine and Jiangsu Key Laboratory of Molecular Medicine, Medical School, Nanjing University, Nanjing, 210093 Jiangsu China; 2 Department of Clinical Laboratory, Affiliated Hospital of Integrated Traditional Chinese and Western Medicine, Nanjing University of Chinese Medicine, Jiangsu Province Academy of Traditional Chinese Medicine, Nanjing, 210028 China; 3 Department of Clinical Laboratory, Affiliated Hospital of Integrated Traditional Chinese and Western Medicine, Jiangsu Province Academy of Traditional Chinese Medicine, Nanjing, 210028 China

**Keywords:** Intestinal inflammation, Intestinal mucosal barrier, Light-induced sleep disorder, Puerarin

## Abstract

More and more evidence suggests that puerarin, a potential remedy for gut inflammation, may have an ameliorative effect on sleep disturbances. However, the relationship between puerarin and sleep disruption has not been extensively researched. This study aims to explore the role and mechanisms of puerarin in improving sleep disorders. We established a light-induced sleep disorder model in mice and assessed the effects of puerarin on cognitive behavior using open field and water maze tests. Pathological detection demonstrated that sleep disturbances resulted in observable damage to the liver, lung, and kidney. Puerarin reversed multi-organ damage and inflammation. Further, puerarin activated paneth cells, resulting in increased lysozyme and TGF-β production, and stimulating intestinal stem cell proliferation. Puerarin also effectively inhibited the expression of F4/80, iNOS, TNF-α, and IL-1β in the small intestine, while it increased Chil3, CD206, and Arg-1 levels. Moreover, puerarin treatment significantly decreased P-P65, TLR4, Bcl-xl, and cleaved caspase-3 protein levels while increasing barrier protein levels, including ZO-1, Occludin, Claudin 1 and E-cadherin suggesting a reduction in inflammation and apoptosis in the gut. Overall, puerarin diminished systemic inflammation, particularly intestinal inflammation, and enhanced intestinal barrier integrity in mice with sleep disorders. Our findings suggest a potential new therapeutic pathway for sleep disorders.

**Graphical Abstract:**

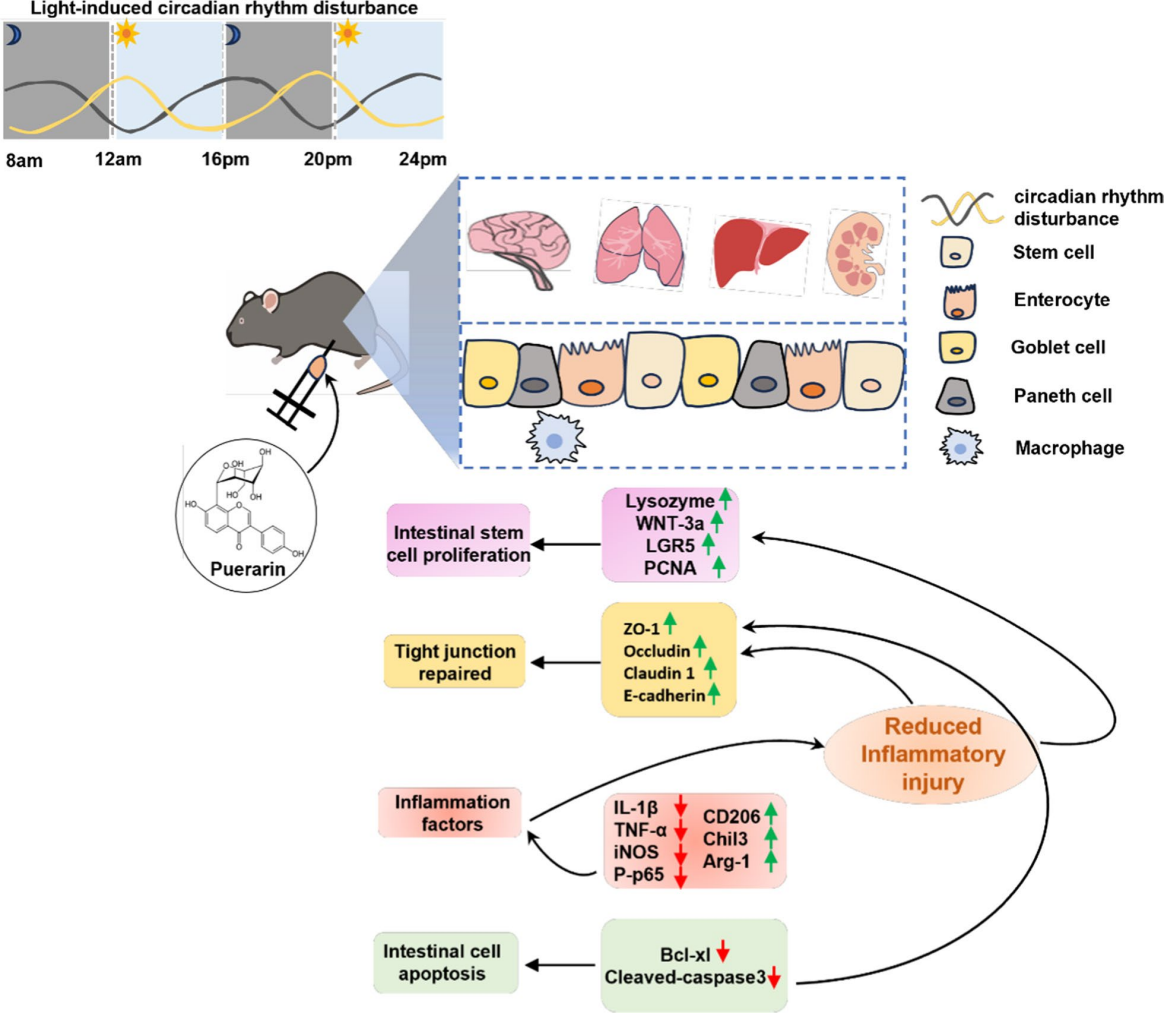

## Introduction

Sleep disorders are highly prevalent among elderly individuals, typified by persistent and recurring difficulties in initiating or maintaining sleep, culminating in inadequate sleep quality. Studies have reported that a considerable proportion of individuals between 60 and 90 years of age, approximately 80–90%, suffer from sleep disorders [1]. These disorders represent a common health concern within this demographic, with their adverse effects on daily activities and overall health well established [2]. Emerging evidence suggests a link between a decline in intestinal barrier function and sleep disorders in the elderly [3]. Existing therapeutic drugs for sleep disorders often come with side effects and safety issues [4]. Therefore, preserving and restoring the intestinal barrier could provide a beneficial strategy for mitigating sleep disorders among older adults [5].

Puerarin is a natural phytonutrient rich in isoflavones, which can improve blood circulation and appetite, relieve sleep disorders, and enhance human vitality [6]. Mood disorders are common mental health issues that affect sleep. Depression is one of the important causes of sleep disorders in the elderly [7]. Studies have shown that puerarin alleviates depressive symptoms in ovariectomized female rats by regulating the hippocampal cAMP-CREB-BDNF signaling pathway [8]. Further studies have shown that the use of Chinese herbal medicine synergistic treatment (such as puerarin) in patients with insomnia can reduce the risk of depression in patients with insomnia and play a role in improving sleep [9]. At the same time, experimental results show that puerarin fermented liquid has good biological activity, which is beneficial to improve the subjective symptoms of insomnia in the elderly population [10]. Not only that, but puerarin, a naturally derived plant nutrient, plays a unique role in protecting and revitalizing the integrity of the intestinal barrier [11]. As a polyphenolic compound, it helps reduce intestinal inflammation, enhance instestinal mucosal barrier function, strengthen immune response, and promote overall health [12, 13]. Although prior studies have proposed that puerarin may improve sleep [14], the specific mechanism involved in sleep disorders has yet to be clearly defined. Thus, this study aims to elucidate the mechanistic aspects of puerarin-mediated intestinal barrier repair with the goal of ameliorating sleep disturbances in the geriatric population.

In our study, senile mice are divided into four groups: the normal control group (NC), the light-induced sleep disturbance group (LSD), the sleep disturbance group treated with puerarin (LP), and the puerarin-only group (PUE). The experimental period spans four weeks, during which we will assess the spatial learning and memory capabilities of the mice, multi-organ pathological manifestations, and markers of intestinal inflammation and barrier function. We posit that this investigation will offer novel insights into the mechanisms through which puerarin enhances the intestinal barrier, ultimately contributing to the management of sleep-related issues in the geriatric population.

## Results

### Puerarin ameliorates cognitive impairment in senile mice with sleep disturbance

**Fig. 1:**
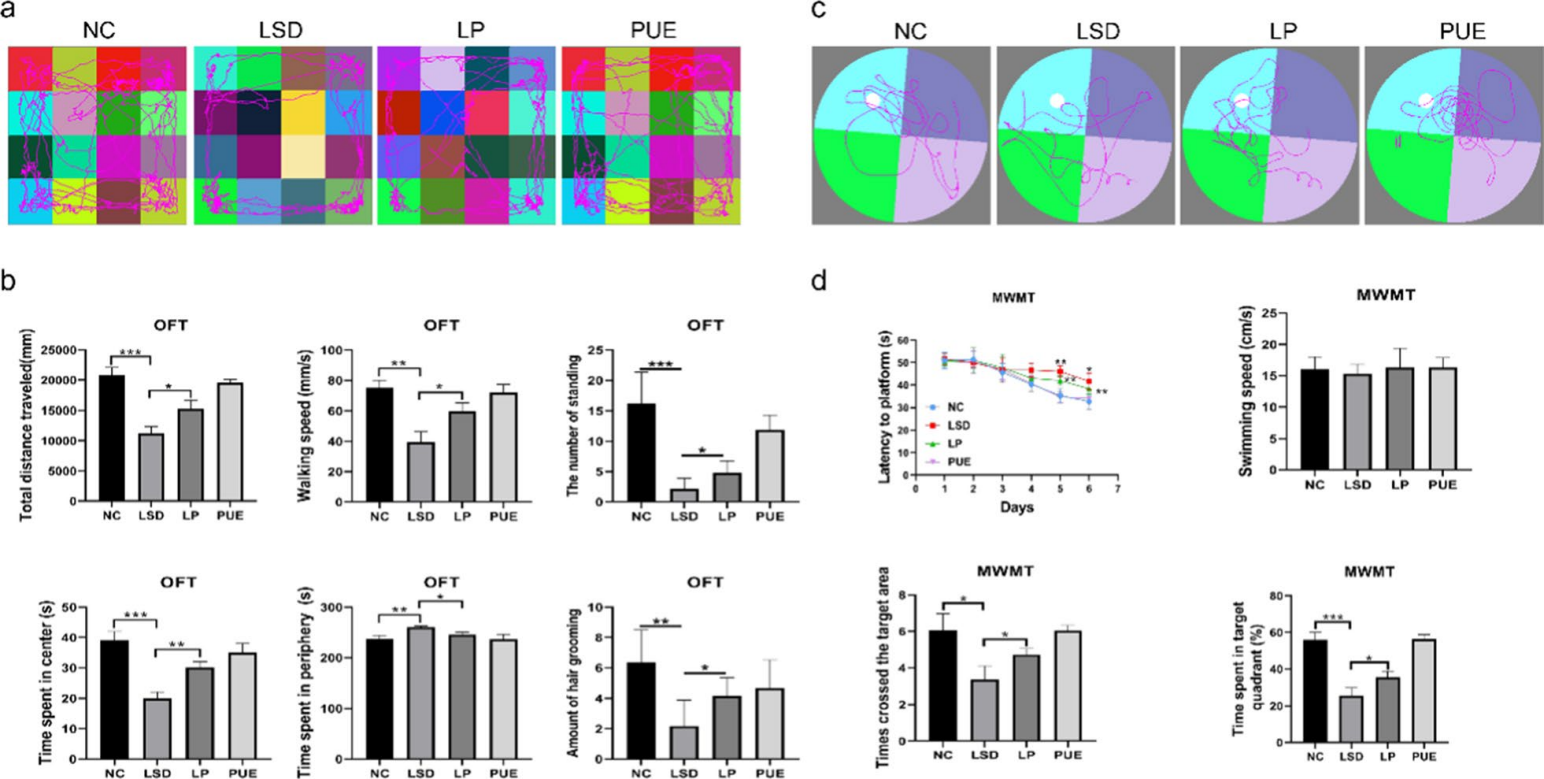
Puerarin attenuated cognitive deficits induced by sleep disturbance. **a** Mice trajectory in the open field experiment. **b** Analysis of total distance, speed, the number of standing, time spent in central and peripheral areas, and the amount of hair grooming in the open field test. **c** Track map of the mouse water maze test. **d** Analysis of escape latency, swimming speed, number of target quadrant crossings, and time spent in the target quadrant in the water maze test. Data are represented as mean ± SD from multi-group experiments. *p < 0.05, **p < 0.01, and ***p < 0.001 (n = 6 each group)

Sleep disturbances are widely acknowledged as risk factors for cognitive decline and various health issues in elderly individuals [15]. In our study, we utilized light modulation to induce sleep disturbances in senile mice, thereby simulating alterations in their circadian rhythms. Notably, in the open field test, we observed a substantial decrease in key parameters including total distance traveled, velocity, the number of standing, the amount of hair grooming and duration in the central area in mice with LSD, as they spent more time in the periphery. Conversely, administration of puerarin led to a notable increase in these parameters (Fig. 1a, b), suggesting its positive impact on locomotor activity and exploratory tendencies in sleep-disturbed mice. In the Morris water maze test, we noted that by the fifth experimental day, the sleep disorder group had significantly longer escape latencies than the control group, a phenomenon mitigated by puerarin. Furthermore, LSD mice crossed the target quadrant less frequently and spent less time there, whereas puerarin administration resulted in increased time spent in this quadrant. However, no statistical difference was observed in the swimming velocities of the mice (Fig. 1c, d). Our results hence suggest that puerarin improves spatial recognition and memory capabilities in LSD mice.

### Effects of puerarin on systemic pathology of sleep disorders in the elderly

**Fig. 2:**
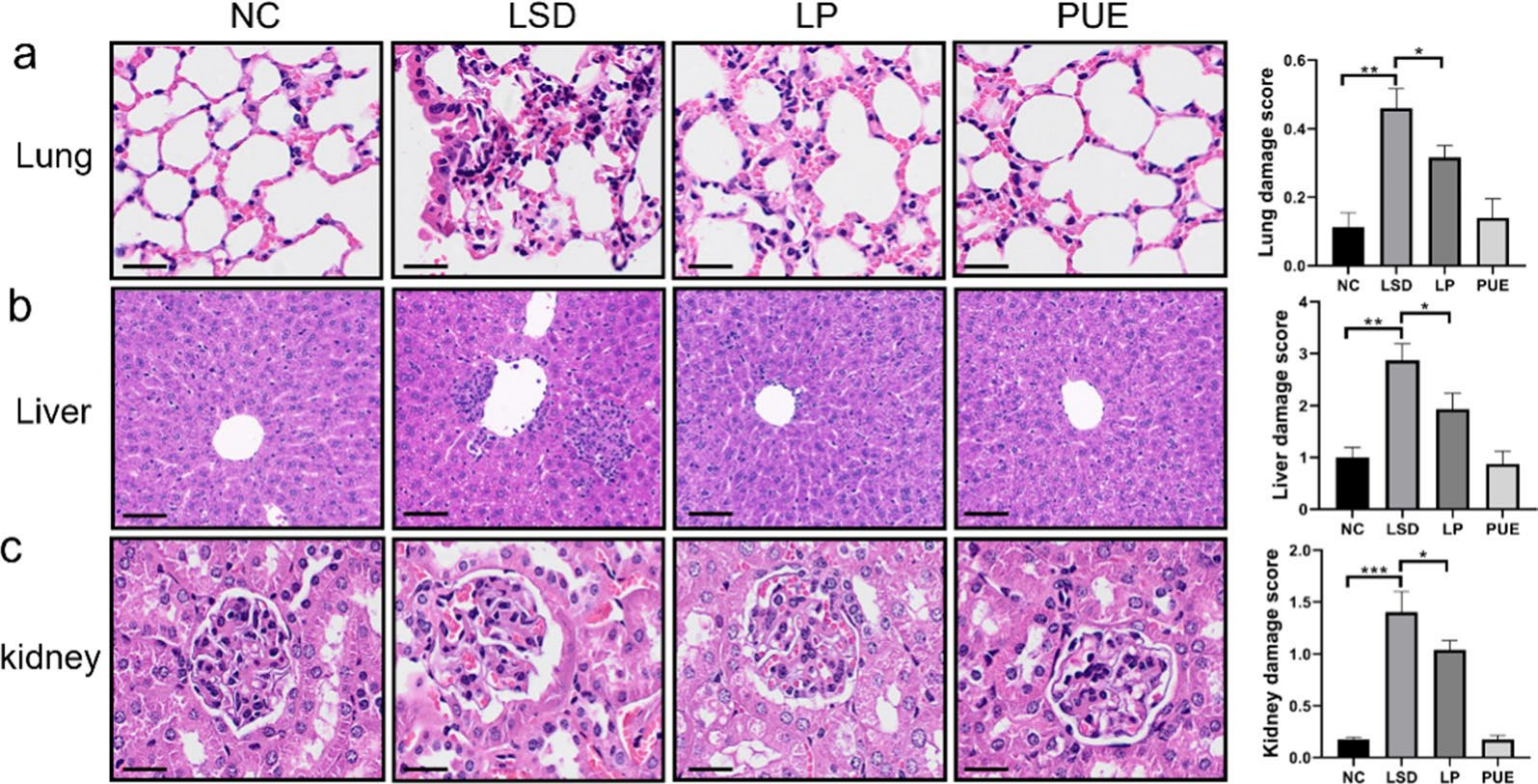
Effects of puerarin on multiple organ pathology in senile sleep disorders. **a** Lung tissue section was stained with hematoxylin and eosin and displayed with quantitative analysis. **b** Liver tissue section was stained with hematoxylin and eosin and analyzed with quantitative analysis. **c** Kidney tissue section was stained with hematoxylin and eosin and displayed with quantitative analysis. Data represent mean ± SD from multi-group experiments. *p < 0.05, **p < 0.01, and ***p < 0.001 (n = 6 each group)

In addition to cognitive-behavioral effects, sleep disorders in the elderly have been associated with varying degrees of damage to multiple organs throughout the body [16]. We explored the pathological states of the lungs, liver, and kidneys using hematoxylin and eosin (H&E) staining. Our findings revealed significant disruption of the lung tissue structure with an accumulation of inflammatory cells surrounding the alveoli in LSD mice. Conversely, puerarin treatment led to partial restoration of the alveolar structure and a reduction in inflammatory cell count (Fig. 2a). Furthermore, we observed infiltration of inflammatory cells in the liver of LSD mice; this infiltration was noticeably reduced post-puerarin treatment (Fig. 2b). An examination of the kidneys using H&E staining showed ruptured tubular epithelial cells, expanded Bowman’s space, dilated tubular lumens, and slight capillary bruising in the LSD mice. Puerarin treatment, in contrast, resulted in a reduction in Bowman’s space and alleviated capillary congestion (Fig. 2c). In summary, puerarin exhibited notable anti-inflammatory properties and ameliorated the multi-organ pathological damage induced by sleep disturbance.

### Puerarin attenuates intestinal pathological changes in elderly mice with sleep disturbances

**Fig. 3:**
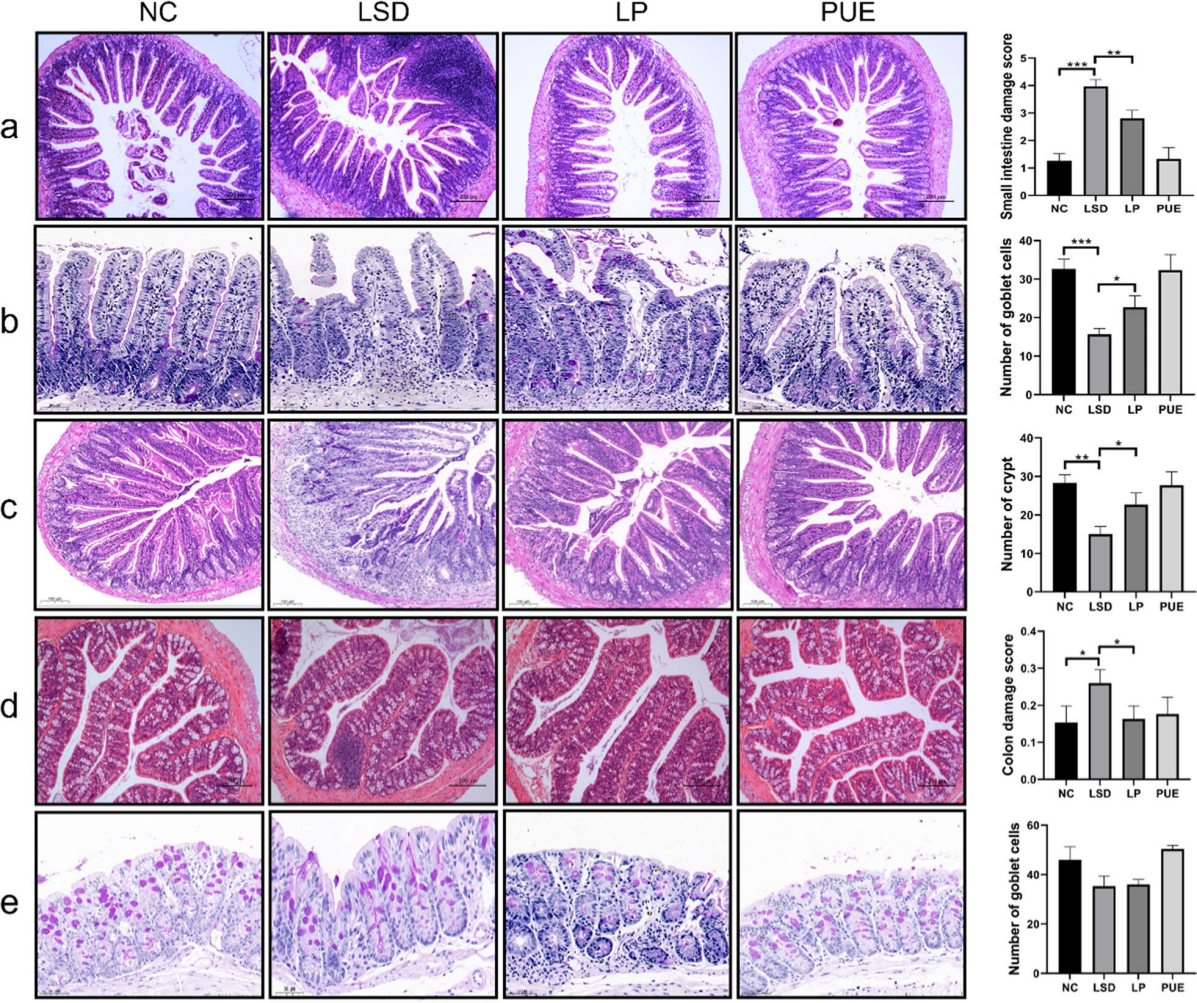
Puerarin improved the microscopic structure of intestinal tissue. **a** Small intestine tissue section was stained with hematoxylin and eosin and displayed with quantitative analysis. **b** Goblet cells in small intestine were evaluated by periodic acid-Schiff staining with quantitative analysis of cell number. **c** Paneth cells in small intestine was detected with Phloxine B and the number of crypts was analyzed. **d** Colon section was stained with hematoxylin and eosin and displayed with quantitative analysis. **e** Goblet cells in colon were evaluated by periodic acid-Schiff staining with quantitative analysis of cell number. Data represent mean ± SD from multi-group experiments. *p < 0.05, **p < 0.01, and ***p < 0.001 (n = 6 each group)

The small intestine, alongside the lungs, liver, and kidneys, functions as a vital immune organ [17]. Initial examinations of histopathological alterations in small intestinal tissues via H&E staining revealed marked inflammatory cell infiltration and disrupted crypt architecture. Puerarin treatment markedly reduced inflammation in the small intestine and significantly reinstated crypt integrity (Fig. 3a). Goblet cells, essential intestinal secretory cells, secrete mucin to maintain the intrinsic intestinal barrier [18]. PAS staining revealed a significant reduction in goblet cell count in the small intestine of LSD mice relative to controls, whereas puerarin treatment led to an increase in goblet cell count relative to LSD mice (Fig. 3b). This suggests that sleep disorders may compromise the integrity of the small intestine’s barrier function. Paneth cells are pivotal for sustaining innate immunity and contributing to antibacterial functions in the gut [19]. Phloxine B staining illustrated disrupted crypt structure and a notable decrease in Paneth cell count in LSD mice. Post-puerarin treatment, both the crypt architecture and Paneth cell count were notably restored (Fig. 3c). We also observed partial changes in the colon during sleep disturbance. The colon exhibited inflammatory cell infiltration, albeit less so than the small intestine, as evidenced by H&E staining. Puerarin exerted anti-inflammatory effects in the colon of LSD mice (Fig. 3d). Nevertheless, no significant changes in colonic goblet cell count were detected among all groups (Fig. 3e), which seems to indicate from another perspective that sleep disturbance affects the barrier function of the colon to a lesser degree than that of the small intestine.

### Puerarin modulates paneth cell function and enhances intestinal stem cell proliferation

**Fig. 4:**
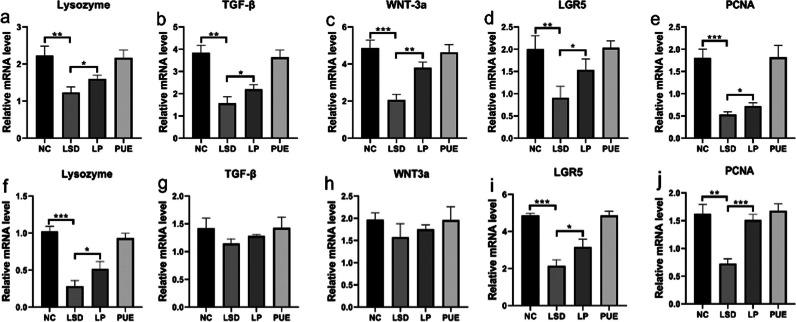
Puerarin acts on Paneth cells to promote proliferation of intestinal stem cells. **a–e** Small intestine RNA was extracted and the expression of lysozyme, TGF-β, WNT3a, LGR5, and PCNA was analyzed by qRT-PCR. **f**–**j** Colon RNA was extracted and the expression of lysozyme, TGF-β, WNT3a, LGR5, and PCNA was analyzed by qRT-PCR. Data represent mean ± SD from multi-group experiments. *p < 0.05, **p < 0.01, and ***p < 0.001 (n = 6 each group)

Beyond its role in mitigating structural abnormalities within intestinal tissue induced by sleep disturbances, puerarin also affects the functionality of intestinal cells. The importance of Paneth cells and intestinal stem cells in intestinal function is well established [20, 21]. Initially, we evaluated lysozyme expression, primarily derived from Paneth cells, in the small intestine using qPCR. The observed decrease in lysozyme levels amid sleep disturbances (Fig. 4a) suggested a reduction in the intestinal antibacterial and anti-inflammatory capabilities, which were notably restored with puerarin administration. Subsequently, a marked decrease in TGF-β levels in the small intestine was noted during sleep disturbances, indicating a diminished anti-inflammatory response. Nevertheless, puerarin treatment significantly augmented TGF-β levels (Fig. 4b). Further, LGR5, an established marker of adult intestinal stem cells [22], demonstrated a significant reduction per qPCR results, suggesting a decline in intestinal stem cell count during sleep disturbances (Fig. 4c). Expression levels of PCNA and Wnt3a, both indicative of cell proliferation, were also reduced in sleep disturbances [23]. However, post-puerarin treatment, their expression levels markedly increased in sleep-disordered mice (Fig. 4d-e). In a similar manner, puerarin was observed to exert the same modulatory effect on colonic inflammation and intestinal stem cells under conditions of sleep disorders, including an increase in lysozyme levels (Fig. 4f) and a promotion of intestinal stem cell proliferation (Fig. 4h–j). Nevertheless, no significant differences were observed in the expression levels of TGF-β and WNT3a genes in the colon.

### Puerarin regulates macrophages to inhibit intestinal inflammation in age-related sleep disorders

**Fig. 5:**
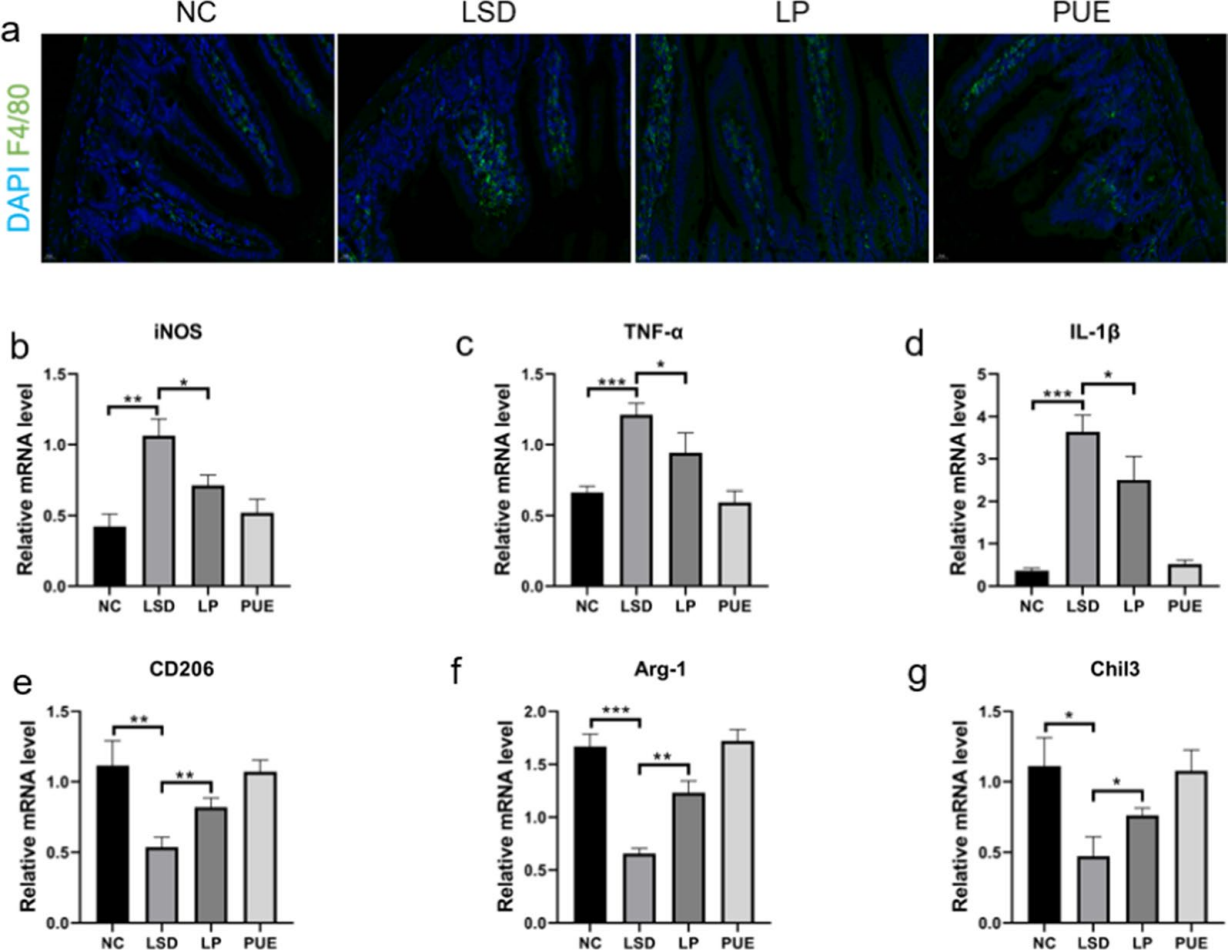
Puerarin reduced intestinal inflammation by modulating macrophages. **a** Representative immunofluorescence image: F4/80 (green) and DAPI (blue). Scale bar = 50 μm. **b–d** Small intestinal RNA was extracted and the expression levels of M1 macrophage markers iNOS, TNF-α, and IL-1β were detected by qRT-PCR. **e–g** Small intestinal RNA was extracted and the expression levels of M2 macrophage markers CD206, Arg-1, and Chil3 were detected by qRT-PCR. Data represent mean ± SD from multi-group experiments. *p < 0.05, **p < 0.01, and ***p < 0.001 (n = 6 each group)

The reconfiguration of macrophage polarization is critical in preserving the homeostasis of the intestinal microenvironment during intestinal mucosal repair [24]. We investigated the effect of puerarin on macrophages, starting with immunofluorescent staining for F4/80. Compared to the negative control group (NC), the sleep disturbance group (LSD) demonstrated a significantly increased expression of F4/80. However, this elevation was notably reduced by puerarin treatment (Fig. 5a). In addition, we quantified the expression of iNOS, TNF-α, and IL-1β, which are established M1 macrophage markers, using qPCR. Our findings revealed that sleep disturbance induced a considerable shift of small intestinal macrophages to the M1 phenotype, which was attenuated by puerarin (Fig. 5b–d). Concurrently, there was a dramatic reduction in the expression of M2 markers CD206, Arg-1, and Chil3. Puerarin, however, enhanced the expression of these M2 markers in LSD mice (Fig. 5e-g). Collectively, these results suggest that puerarin favors the conversion of macrophages from M1 to M2 phenotype, thus conferring anti-inflammatory properties.

### Puerarin inhibits inflammation and apoptosis in intestinal cells and improves intestinal permeability

**Fig. 6:**
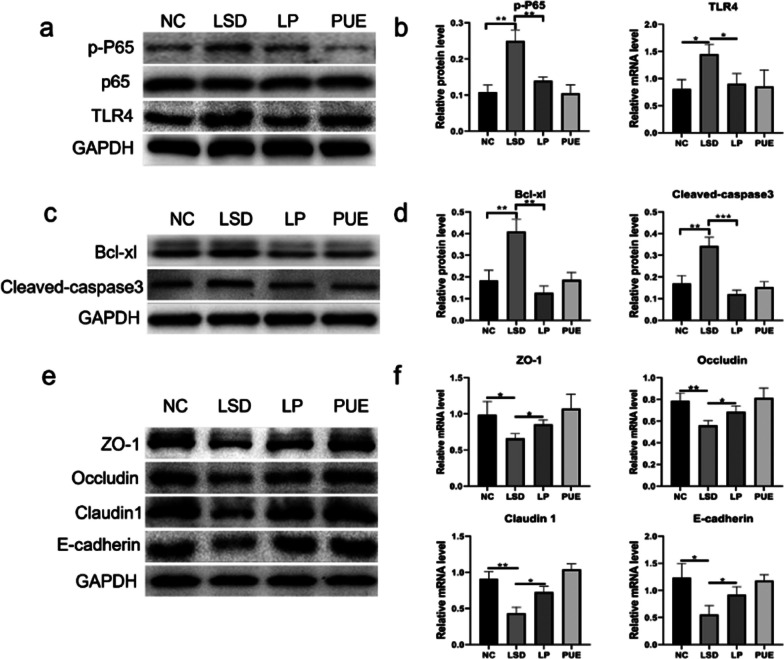
Puerarin improved intestinal permeability by regulating intestinal cell inflammation and apoptosis. **a**, **b** Small intestinal protein was extracted and protein expression levels of p65, p-p65 and TLR4 were detected by Western blotting. **c**, **d** Protein expression levels of Bcl-xl and cleaved caspase-3 in the small intestinal was detected by Western blotting. **e** Protein expression levels of ZO-1, Occludin, Claudin 1, and E-cadherin in the small intestinal were detected by Western blotting. **f** The mRNA expression levels of ZO-1, Occludin, Claudin 1, and E-cadherin in the small intestinal was detected by qRT-PCR. Data represent mean ± SD from multi-group experiments. *p < 0.05, **p < 0.01, and ***p < 0.001 (n = 6 each group)

To further substantiate the role of puerarin on intestinal barrier function, we evaluated the protein expression in the intestine. Our results showed activation of the NF-κB inflammatory signaling pathway and a substantial increase in phosphorylated P65 and TLR4 protein following sleep disturbance (Fig. 6a, b). Concurrently, the apoptotic proteins, Bcl-xl and cleaved caspase-3, were found to be markedly upregulated in the intestine (Fig. 6c, d). Interestingly, puerarin treatment reduced the expression of inflammatory protein P-P65 and apoptotic proteins Bcl-xl and cleaved caspase-3 at protein level, indicating its anti-inflammatory and anti-apoptotic potential. Further investigation revealed a notable augmentation in the expression of intestinal permeability-associated proteins, ZO-1, occluding, Claudin 1 and E-cadherin, post-puerarin treatment, as compared to LSD mice (Fig. 6e, f). Our findings thus suggest that puerarin enhances intestinal permeability, potentially through the attenuation of intestinal inflammation and apoptosis.

## Discussion and conclusion

Recent studies have reported an association between sleep disturbances and puerarin [25]. In our research, we focused on delineating the role puerarin might play in mitigating the effects of light-induced sleep disruptions. Prior work has established memory impairment as a salient indicator of sleep dysregulation in comparison to unaffected individuals [26]. Moreover, studies have found increased intestinal permeability in geriatric patients suffering from sleep disorders [27]. However, the exact function of puerarin in context with these phenomena remains an open question that necessitates additional investigation. In this study, we developed a model of light-induced sleep disturbance using aged mice and noted a significant reduction in their spatial memory and motor skills following the sleep disturbance. However, upon administering puerarin, an improvement in cognitive and physical capabilities was observed, along with a decrease in intestinal permeability. This observation could shed light on a potential mechanism through which puerarin ameliorates cognitive impairments related to sleep disturbances.

Sleep has long been recognized as an essential component in brain maturation, as well as in the development and maintenance of cognitive functions [28, 29]. Our findings indicate that puerarin treatment markedly improved cognitive behavior and spatial motor skills in mice with light-induced sleep disorders. This aligns with previous studies that demonstrated puerarin’s ability to mitigate neuronal cell loss in a rat model of cerebral ischemia-induced vascular dementia [26]. Sleep disturbances have been acknowledged as exacerbating factors for patients suffering from various diseases. They influence the pathophysiology of several organ systems, including the lungs, liver, kidneys, and intestines [27, 30, 31]. More specifically, sleep disturbances exacerbate lipopolysaccharide (LPS)-induced acute lung inflammation, which is characterized by inflammatory cell infiltration, increased apoptosis, and membrane hyperoxidation [32]. Even minor sleep deprivation can modify molecular processes, resulting in cellular immune activation and the induction of inflammatory cytokines [33]. A cohort study further underscored this relationship, revealing that compared to a healthy control group of 69,496 individuals, 17,374 patients with obstructive sleep apnea exhibited more than a fivefold increased risk of liver disease, particularly liver cirrhosis and hepatitis C [34, 35]. Our study, through H&E staining, showed that light-induced sleep disturbance can precipitate liver damage, accompanied by inflammatory cell infiltration at the injury site. Moreover, mounting evidence suggests that sleep disturbances influence the progression of kidney disease, potentially due to an inflammatory environment and sympathetic activation in the renal vascular bed, which damages the glomerular basement membrane [36]. Our H&E staining results revealed that mice with light-induced sleep disorders exhibited congested renal microvessels, enlarged Bowman’s space, and a disrupted basement membrane. However, our study also demonstrated that following puerarin treatment, the extent of damage and the inflammatory state in multiple organs (lung, liver, and kidney) was significantly reduced, confirming the potent anti-inflammatory properties of puerarin.

The established relationship between sleep disruptions and gastrointestinal (GI) disorders has been well-documented in recent studies [37–40]. It has been shown that sleep deprivation can intensify GI symptoms, while a plethora of GI disorders can adversely impact the sleep-wake cycle [41, 42]. More specifically, sleep disturbance-induced pathological damage, manifested as changes in crypt structure, epithelial cell injury, and inflammatory cell infiltration, is more pronounced in the small intestine compared to the colon. Intriguingly, our study found that treatment with puerarin not only accelerated the recovery of damaged intestinal tissue structure but also elevated the count of goblet and Paneth cells in the small intestine. Goblet cells, which are found throughout the small and large intestines, manufacture and secrete high molecular weight glycoproteins known as mucins, responsible for forming and maintaining a protective mucus layer [43]. Paneth cells, on the other hand, predominantly produce antimicrobial proteins that are believed to influence the composition and abundance of native gut microorganisms [44]. These findings suggest that puerarin might augment intestinal barrier function and antibacterial efficacy by increasing the production of goblet and Paneth cells. Of note, sleep disturbances appeared to have a lesser effect on the colon, as evidenced by only a minor variation in the number of goblet cells in colonic tissue.

Moreover, the disruption of the gut barrier has been strongly associated with inadequate sleep [45]. When the integrity of the gut barrier is compromised, harmful substances such as bacteria and toxins can infiltrate the bloodstream, instigating an inflammatory response [46, 47]. In parallel, sleep disturbances can augment gut permeability and deplete the population of beneficial gut bacteria [48], potentially leading to inflammation and a range of negative health outcomes. Intestinal stem cells play a crucial role in maintaining the integrity of the gut barrier through the continual regeneration of the epithelial lining and the repair of any damage [49].

Furthermore, Paneth cells have been noted to secrete Wnt ligands, which consequently activate the Wnt signaling pathway in intestinal stem cells [50]. In this investigation, we observed that puerarin was able to augment lysozyme levels in cases of sleep disruption. This suggests that puerarin might stimulate an increase in Paneth cell numbers or boost their secretory function. Our data further substantiates that puerarin administration led to an elevation of Wnt ligand Wnt3a levels in the gut, alongside a discernible trend for LGR5 and PCNA. As Lgr5 serves as a marker for human intestinal stem cells and increased levels of PCNA signify cell proliferation [51], our findings suggest that puerarin could potentially activate intestinal stem cell proliferation through regulation of Paneth cell function, hence enhancing intestinal barrier function. Additionally, intestinal barrier function can be fortified by inhibiting intestinal epithelial cell apoptosis [52]. The expression of Bcl-xl and cleaved caspase-3 proteins was notably higher in the group with sleep disturbances compared to the NC group, indicating that sleep disturbances induce apoptosis of intestinal epithelial cells. Decreased levels of ZO-1, Occludin, Claudin 1 and E-cadherin proteins suggest that sleep disturbances might increase intestinal permeability. However, our data revealed that puerarin could inhibit the apoptosis of intestinal epithelial cells and diminish intestinal permeability.

Sleep disorders have been found to impact bodily immune functions, particularly affecting intestinal inflammation [53]. Our results show that the small intestine of LSD mice exhibits significantly higher protein levels of p-p65 and TLR4 compared to the NC group mice. This elevation was curtailed by puerarin treatment. Our study thus suggests that puerarin demonstrates an anti-inflammatory effect on the intestinal tract by reducing the number of macrophages and promoting a shift from M1 to M2 macrophage phenotype. Such an inflammatory state within the intestine could possibly alter the composition of intestinal microorganisms and intestinal metabolites, impacting brain function via the gut-brain axis, which may influence sleep quality [54]. Therefore, puerarin might improve sleep quality by modulating intestinal macrophages and the overall intestinal inflammatory state.

In conclusion, our study provides evidence suggesting that puerarin can alleviate sleep disturbances in aged, sleep-disordered mice by improving intestinal barrier function and reducing intestinal inflammation. Additionally, puerarin was found to ameliorate cognitive and memory impairments in mice, mitigating the adverse effects of sleep disturbances. Considering puerarin is rapidly cleared from circulation and exhibits low toxicity, further preclinical studies exploring its potential therapeutic application in sleep disorders are warranted [55].

## Materials and methods

### Experimental animal procedure

Male C57BL/6J mice (Specific Pathogen Free, SPF), 52 weeks old, average weight 35 g, were obtained from Model Animal Genetics Research Center of Nanjing University (Nanjing, China). The mice were given a one-week acclimatization period with ad libitum access to food and water in environment at a temperature of 22 ± 2 °C, with a relative humidity of 50 ± 1% to being divided into four distinct experimental groups. Zeitgeber time (ZT), a measure based on predictable light-dark cycles, was employed as the uniform temporal benchmark. ZT0 was designated as the onset of the light phase at 8:00, while ZT12 marked the beginning of the dark phase at 20:00. The control group (NC group), was subjected to a light phase from ZT0 to ZT12, followed by a dark phase from ZT12 to ZT24. The light-induced sleep disturbance group (LSD group), experienced a randomized light phase delay of 0–4 h, while preserving a 12-hour duration for both light and dark phases. The light intensity in the lit section was regulated at 800 lx to prevent the onset of circadian arrhythmias. In the third group, the LSD + puerarin group (LP group), mice were administered intraperitoneal injections of puerarin (160 mg/kg). A puerarin-only group (PUE) was subjected to the same treatment. The experimental model was conducted over a period of one month.

### Behavioral assays

#### Open field test

We utilized an open field apparatus constructed from white polymethyl methacrylate, measuring 40 cm in each dimension. Each mouse was initially positioned in the center of the apparatus, and their movements were subsequently tracked and recorded over a period of five minutes with the assistance of a camera. The obtained data was then subjected to analysis via the TopsanLite 2.0 software, enabling the evaluation of various parameters such as the total distance covered, speed, the number of standing, the amount of hair grooming and time duration spent in the central or peripheral zones of the apparatus.

#### Morris water maze test

In order to evaluate the cognitive capabilities of the mice, we employed the Morris water maze test (MWMT). The MWMT setup encompasses a circular pool (120 cm in diameter and 50 cm in height), an escape platform with a diameter of 12 cm, and an image capture and analysis software supplied by the Yi Shu Information Technology Co. Ltd., Shanghai, China. The experiment was bifurcated into two segments: the hidden platform trial and the probe trial. The hidden platform trial spanned over a period of five successive days. During this trial, each mouse was introduced into a randomly chosen quadrant and given a maximum of 60 s to locate the submerged platform, which was positioned 2 cm below the water surface. Upon locating the platform, the mouse was allowed to stay on it for a period of 20 s. This process was repeated four times a day for each mouse, with an intermission of 30 s between each trial. During each trial, the mouse was positioned in the pool, facing the wall, in a fixed quadrant. The trial either concluded when the mouse located the platform or when the allotted time of 60 s was reached. In cases where the mouse was unable to locate the platform within the given time, it was guided to the platform by a technician and allowed to stay there for 15 s. The time required by the mouse to reach the platform was recorded and termed as the escape latency. On the sixth day, the probe trial was conducted, during which the hidden platform was removed. The mice were allowed to swim freely for 60 s. Metrics such as the frequency of a mouse crossing the former platform area and the time duration spent in the target quadrant were recorded, serving as measures of spatial memory.

### Hematoxylin and eosin, periodic acid-Schiff, and phloxine B staining

The collected tissue samples were first fixed in a 4% paraformaldehyde solution for a period of 48 h. This was followed by a process of dehydration and paraffin embedding. Then, the tissue samples were sectioned at a thickness of 5 μm using a rotary microtome. First, the slices were stained with HE, dewaxing for about 10 min each step, stained with hematoxylin reagent for 5 min, stained with eosin for 1 min, dehydrated in different grades of alcohol for 10 s, xylene for 1 min, and sealed with neutral resin after drying. The morphological characteristics of various tissues—lung, liver, kidney, small intestine, and colon—were then assessed using H&E staining, as provided by Servicebio (Wuhan, China). The histological scores were determined based on the criteria established in previous research [56–58]. Periodic acid-Schiff (PAS) staining was carried out on sections of the small intestine and colon, in accordance with the manufacturer’s protocols (Catalogue No. G1008; Servicebio), to evaluate the mucus layer and goblet cell population. For the specific staining of Paneth cells in the small intestine, 5 μm sections were deparaffinized and rehydrated. The slices were washed twice with distilled water, followed by periodic acid alcohol solution for 10 min. Rinse the slices with distilled water for 10 min, incubate in Schiff’s solution for 10 min, then rinse with running water for 5 min. Subsequently, the nuclei were stained with Harry’s hematoxylin for 3 min, and then washed with running water for 5 min. After a final rinse and mounting, the sections were examined using light microscopy. In this manner, Paneth cell granules were identified as red structures.

### Immunofluorescence assay

Sections of 5 µm thickness were incubated overnight at 4°C with a rabbit anti-F4/80 antibody (Catalogue No. GB11027, Servicebio). Subsequent to this, the sections were exposed to a goat anti-rabbit IgG [H + L] secondary antibody (Catalogue No. GB21303, Servicebio). In the concluding step, sections were stained with a 4’,6-diamidino-2-phenylindole (DAPI) solution (Catalogue No. G1012, Servicebio) for 10 min at room temperature in darkness. Fluorescent images of the treated sections were acquired for further examination using a Nikon Eclipse TI-SR fluorescence microscope.

### Quantitative real-time PCR (RT-qPCR)

RNA was extracted from liver, small intestine, and colon tissues utilizing Trizol reagent. Subsequent measurement of RNA concentration and purity was executed using a NanoDrop2000 spectrophotometer (Thermo Scientific, Shanghai, China). Thereafter, 1 µg of RNA was reverse-transcribed to cDNA employing the Prime Script First Strand cDNA Synthesis Kit. The SYBR Premix Ex Taq kit was used to perform the quantitative real-time polymerase chain reaction (qRT-PCR) on a Light Cycler 480 (Roche, Switzerland) for evaluating the expression levels of *lysozyme*, *TGF-β, WNT-3a, LGR5, PCNA, iNOS, TNF-α, IL-1β, CD206, Arg-1, Chil3*, and *β-actin, ZO-1, Occludin, Claudin1, and E-cadherin*. Thermal cycling conditions were set according to the manufacturer’s directives. Cycle threshold values were computed using the 2^−ΔΔCT^ method to assess relative gene expression levels. The housekeeping gene, *β-actin*, was used as a control for relative quantification of target RNA transcript abundance. All RT-qPCR experiments were performed in triplicate for technical reliability. The sequences of gene-specific primers utilized are provided in Table 1.

**Table 1:** The primers used for qRT-PCR

Primers	Forward primer (5 '-3 ')	Reverse primer (5 '-3 ')
*WNT-3a*	ATTGAATTTGGAGGAATGGT	CTTGAAGTACGTGTAACGTG
*TGF-β*	CTCCCGTGGCTTCTAGTGC	GCCTTAGTTTGGACAGGATCTG
*Lysozyme*	CCTCTGTAGGTCAGTTC	GGATCAACTGGTCTCCTATAA
*LGR5*	GCAACAACAUCAGGUCAAUTT	AUUGACCUGAUGUUGUUGCTT
*PCNA*	GAGAGCTTGGCAATGGGAACA	GGGCACATCTGCAGACATACTGA
*iNOS*	ACTCAGCCAAGCCCTCACCTAC	TCCAATCTCTGCCTATCCGTCTCG
*TNF-α*	ACGGCATGGATCTCAAAGAC	GTGGGTGAGGAGCACGTAGT
*IL-1β*	GCAACTGTTCCTGAACTCAACT	ATCTTTTGGGGTCCGTCAACT
*CD206*	CTCTGTTCAGCTATTGGACGC	TGGCACTCCCAAACATAATTTGA
*Arg-1*	CATATCTGCCAAAGACATCGTG	GACATCAAAGCTCAGGTGAATC
*Chil3*	CTGAATGAAGGAGCCACTGA	AGCCACTGAGCCTTCAACTT
*ZO-1*	TGGTCTGTTTGCCCACTGTT	TCTGTACATGCTGGCCAAGG
*Occludin*	ACGTCCGACCCATGCTCTCT	AAGTCATCCGCAGGGGAGGT
*Claudin1*	TGCCCCAGTGGAAGATTTACT	CTTTGCGAAACGCAGGACAT
*E-cadherin*	CAGTTCCGAGGTCTACACCTT	TGAATCGGGAGTCTTCCGAAAA
*β-actin*	GGACCTGACAGACTACCTCA	TCTTTGATGTCACGCAC

### Western blot analysis

Small intestinal tissue samples (approximately 20 mg) were lysed in an ice-cold RIPA lysis buffer, enriched with protease inhibitors. Subsequent protein concentration measurement was performed using a BCA protein assay kit. Proteins (20 µg per sample) were loaded into 10% polyacrylamide gels, separated through electrophoresis, and then transferred to nitrocellulose membranes. The protein-bound nitrocellulose membranes were blocked with a solution of 5% skim milk in PBS-Tween-20 (0.1%) for two hours at room temperature to avoid non-specific binding. The membranes were incubated with primary antibodies targeting p65 (1:1,000), p-p65 (1:1,000), TLR4 (1:1,000), Bcl-xl (1:1,000), cleaved caspase-3 (1:1,000), ZO-1 (1:1,000), Occludin (1:1,000), Claudin1 (1:1,000), E-cadherin (1:1,000) and GAPDH (1:4,000) at 4 °C overnight. This was followed by an hour-long incubation at room temperature with an HRP-conjugated secondary antibody. An ECL kit was employed to detect specific protein signals. Images of protein bands were captured with a Bio-Rad XRS imaging system and the intensity of the proteins was evaluated semi-quantitatively using ImageJ software.

### Statistical analysis

Except where specifically mentioned in the previous methodological sections, all statistical analyses were conducted using Prism 9 software (GraphPad Software, San Diego, CA). Data are represented as mean ± standard deviation in the form of bar graphs. Student’s *t*-test or the Wilcoxon test was used for the assessment of differences between two groups. For comparisons involving more than three groups, statistical analysis was performed using one-way analysis of variance or the Kruskal-Wallis test. Statistically significant differences are indicated by asterisks as follows: *p < 0.05, **p < 0.01, ***p < 0.001.
